# Agraphia for Kanji Resulting From a Left Posterior Middle Temporal Gyrus Lesion

**DOI:** 10.1155/2008/393912

**Published:** 2008-07-15

**Authors:** Yasuhisa Sakurai, Imari Mimura, Toru Mannen

**Affiliations:** Department of NeurologyMitsui Memorial HospitalTokyoJapan

**Keywords:** Pure agraphia, kanji, kana, alexia with agraphia, posterior middle temporal gyrus, fusiform gyrus, visual word form area

## Abstract

*Objective:* To clarify whether agraphia or alexia occurs in lesions of the left posterior middle temporal gyrus.

*Methods:* We assessed the reading and writing abilities of two patients with this lesion using kanji (Japanese morphograms) and kana (Japanese syllabograms).

*Results:* Patient 1 first presented with pure alexia more impaired for kana after an infarction in the left middle and inferior occipital gyri and right basal occipital cortex, and after a second infarction in the left posterior middle temporal gyrus adjoining the first lesion he showed alexia with agraphia for kanji and worsened alexia for kana; kanji alexia recovered over the following six to 10 months. Patient 2 presented with alexia with agraphia for kanji following a hemorrhage in the left posterior middle and inferior temporal gyri, which resolved to agraphia for kanji at two months after onset. Kana nonword reading was also slightly impaired, but became normal by six months post-onset. In both patients, kanji agraphia was mostly due to impaired character recall.

*Conclusion:* The present patients demonstrate that damage to the left posterior middle temporal gyrus alone can cause agraphia for kanji. If the adjacent mid fusiform/inferior temporal gyri (Area 37) are spared, the kanji alexia is transient.

## 1. Introduction

Agraphia, particularly for kanji, arises from lesions in the posterior inferior temporal cortex [13,16,31, 34], angular gyrus [12,14,20,32], superior parietal lobule [14,18,30] and posterior middle frontal gyrus [28]. A characteristic feature common to all these lesions is impaired character recall or failure to retrieve lexical representations. Since kanji have many irregular readings (described below), agraphia for kanji is comparable to lexical or orthographic agraphia in Western countries [3,34].

Lexical agraphia is due to lesions of the posterior inferior temporal cortex [7,24], angular gyrus [25] and precentral gyrus [23]. Among these lesions, the posterior inferior temporal cortex (more precisely, mid fusiform/inferior temporal gyri, Area 37) is assumed to be the site for an orthographic lexicon [24] or visual word form area (VWFA [5]), where word orthography is accessed. It was suggested that damage to VWFA or the occipitotemporal cortex yields pure alexia [6,22]. It should be noted, however, that a lesion in this area per se does not cause pure alexia, but alexia with agraphia for irregular words and letter-by-letter reading [24] or its Japanese equivalent alexia with agraphia for kanji [26]. In support of this view, a study on patients with acute ischemic stroke revealed that VWFA dysfunction was associated with impairment on all reading, naming and writing tasks that required lexical output [11]. On the other hand, pure alexia occurred in a circumscribed lesion located more medially in the mid fusiform gyrus [33].

We here describe the kanji and kana writing systems briefly. The Japanese language has two distinct writing systems; i.e. kanji (morphograms, originally adopted from Chinese characters) and kana (Japanese phonetic writing or syllabograms, originally taken from

ISSN 0953-4180/08/$17.00 © 2008 – IOS Press and the authors. All rights reserved

Table 1
The WAB test scores

| Patient | Patient 1 | | Patient 2 |
|---|---|---|---|
| Time<br>(after onset, mo.) | June 2005<br>5[a] | July 2005<br>1[b] | Mar 2007<br>2 |
| Spontaneous speech | | | |
| Information content | 10/10 | 9/10 | 10/10 |
| Fluency | 10/10 | 10/10 | 10/10 |
| Naming total | 9.5/10 | 8.6/10 | 9.9/10 |
| Object naming | 58/60 | 55/60 | 60/60 |
| Repetition | 10/10 | 10/10 | 10/10 |
| Comprehension total | 9.15/10* | 9.1/10* | 10/10 |
| Reading total | 10/10 | 8.4/10 | 10/10 |
| Comprehension of sentences | 40/40 | 34/40 | 40/40 |
| Recognition of orally spelled kanji | 6/6 | 2/6 | 6/6 |
| Oral spelling of kanji characters | 6/6 | 0/6* | 6/6 |
| Writing total | 10/10 | 9.7/10 | 9.5/10 |
| Copying | 10/10 | 10/10 | 10/10 |
| Kanji writing from dictation | 6/6 | 4.5/6 | 5/6 |
| Kana writing from dictation | 6/6 | 6/6 | 6/6 |

[a]Five months after the first infarction in the bilateral occipital lobe.
[b]Actually eight days after the second infarction in the posterior middle temporal gyrus.
*More than 2SD below the normal mean [35].

kanji characters). Kana are further divided into hiragana (a cursive form of kana that is used for grammatical formatives), e.g. しかし ([shikashi], however), and katakana (a square form of kana that is used primarily for representing loan words), e.g. カメラ ([kamera], camera). One feature that differentiates Japanese from English is that reading and writing errors occur at a single character level (for phonological and visual errors) as well as at a word level (for semantic errors; see the footnote to Table 3 for error examples). Kanji are graphically complicated and have meanings with two ways of being read: the *on*-reading that conveys the phonetic value and the *kun*-reading that conveys the meaning. Each kanji character has a few *on*-readings and a few *kun*-readings. Kanji words comprise from one to five kanji characters and usually have one reading attached to the meaning. As there are many homophones, particularly in the *on*-reading of kanji characters or words, errors in writing contain homophone errors (see the legend for Fig. 3) that may also be observed in Western countries. Two-character kanji account for the majority of kanji words. There are many more two-character words with *on*-reading than with *kun*-reading. Therefore, normal Japanese readers try to pronounce an unknown two-character kanji word with a typical *on*-reading of the constituent kanji characters. Although how a kanji character is pronounced is thus statistically predicted [10], there is actually no rule for pronouncing a kanji character in a word. This multiplicity in reading makes a number of kanji words comparable to irregular or inconsistent words in Western countries, and causes *on-kun* confusion errors in which words are, similar to English regularization errors, incorrectly but legitimately pronounced, e.g. 相手 ([soushu], incorrect *on*-reading) for 相手 ([aite], correct *kun*-reading, partner). In contrast, kana are graphically simple and have one definite phonetic value and no intrinsic meaning. A kana character represents a mora of spoken Japanese (a single vowel or consonant-vowel combination), which always has the same pronunciation regardless of the position in a word. A kanji word can be transcribed into kana character sequences (kana word) that represent the pronunciation, e.g. 相手 → あいて ([aite], partner).

Due to the use of these dual writing systems, reading and writing impairments in Japan can present as dissociative disturbances between kanji and kana. Agraphia, particularly for kanji as described above, is one example. On the basis of the case studies, some cognitive models of reading and writing in the Japanese language [13,15,26,28] have been proposed in line with the dual-route hypothesis in English. We developed an anatomically constrained dual-route model of writing (Fig. 1) in which orthographic information of a word goes from the posterior inferior temporal cortex (a site for the orthographic lexicon) and proceeds upward under the angular gyrus and superior parietal lobule to travel to the frontal motor and premotor area (orthographic route) whereas phonological information of a word goes from the posterior superior temporal gyrus (a site for the phonological lexicon) to the angular and supramarginal gyri and joins the arcuate fasciculus to travel to the motor and premotor area (phonological route) [28,30]. The orthographic lexicon and the phonological lexicon have a reciprocal connection. According to this hypothesis, kanji characters that are graphically complex depend more on the orthographic route; thus, kanji agraphia results from damage to any part of the orthographic route [30]. In contrast, kana characters that are graphically simple and are directly linked to phonemes depend less on the orthographic route; hence, kana agraphia (impaired kana character recall) occurs less frequently in lesions of the orthographic route. Instead, kana paragraphia (substitution of other kana characters) results from some parts of the phonological route [28,37].

An unsolved problem is whether pure agraphia (without alexia) occurs in a lesion at or around VWFA. In fact, some investigators reported pure agraphia for kanji resulting from nearly the same lesion [34,39]. However, it remains unknown whether there is an anatomical difference that causes alexia with agraphia and pure agraphia for kanji. Here, we report two patients who showed agraphia for kanji after damage to the left posterior middle temporal gyrus and discuss the role of this area in writing.

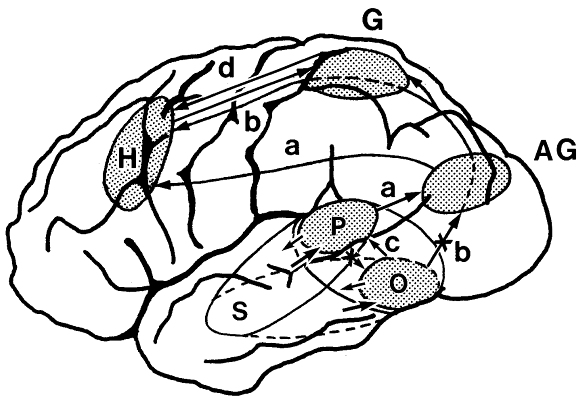

Fig. 1. Diagram of an anatomically constrained dual-route model of writing in response to dictation. a. phonological route, b. orthographic route, c. interaction between phonology and orthography, d. interaction between parietal graphemic area and frontal hand area. P. phonological lexicon, O. orthographic lexicon, S. semantic storage site, AG. angular gyrus, G. graphemic area, H. premotor hand area. Orthographic information of a word goes from the posterior inferior temporal cortex (a site for the orthographic lexicon, O; Area 37) and proceeds upward under the angular gyrus and the superior parietal lobule to travel to the premotor hand area (H; Areas 44/45 and 6). This orthographic route (b) goes directly or indirectly to the hand area via the parietal graphemic area (G), where visuokinesthetic and sequential motor engrams for letters and words are stored. Phonological information of a word goes from the primary auditory cortex (Heschl's gyri) and the posterior superior temporal gyrus (a site for the phonological lexicon, P; Area 22) to the angular and supramarginal gyri and joins the arcuate fasciculus to travel to the frontal motor and premotor areas (a, phonological route). The orthographic lexicon (O) and phonological lexicon (P) have a reciprocal connection (c). Lexico-semantic information (S) is stored in extensive areas in the left temporal lobe, and can be accessed either through the posterior superior temporal gyrus during listening or through the posterior inferior temporal cortex during reading (illustrated by dotted lines). The parietal graphemic area and the frontal hand area (H) have a reciprocal connection (d). Putative damaged routes for kanji agraphia from the posterior middle temporal gyrus lesion are illustrated with "x." Revised from Sakurai et al. [30].

## 2. Materials and methods

### 2.1. Patient profiles

#### 2.1.1. Patient 1

A 75-year-old right-handed man, university graduate and company executive, became aware of blurred vision and felt difficulty reading newspapers in January, 2005. He consulted the department of medicine at our hospital and was diagnosed with a cerebral infarction in the left lateral occipital gyri and the right basal occipital cortex on MRI (Fig. 2a). There were also high intensity areas in the white matter above the two lesions (Fig. 2a–F). Neurological and neuropsychological examinations revealed right homonymous hemianopia (later confirmed by Goldmann perimetry) and alexia, particularly for kana. The patient's clinical data will be included in our research paper on pure alexia for kana. The Western Aphasia Battery (WAB; Japanese edition) administered five months after onset showed a minimal impairment in auditory comprehension (Table 1). Reading and comprehension of sentences were perfect, although he misread some hiragana characters. In July (six months after onset), when reading ability had recovered to within the normal range so that he could read newspapers, he felt faint during writing and could not fill in an invoice. The next day, he found he could not recall or write kanji characters or his son's name. A neuropsychological examination showed alexia with agraphia, particularly for kanji and anomia. He could read two of three two-character kanji words, but could not write the same three kanji words at all. He named correctly only one of six objects visually presented. The patient was admitted to our hospital with a diagnosis of cerebral infarction in the left posterior middle temporal gyrus on MRI. WAB eight days after onset showed slight impairments in auditory comprehension, reading and writing (Table 1). He read sentences slowly and phrase by phrase, sometimes

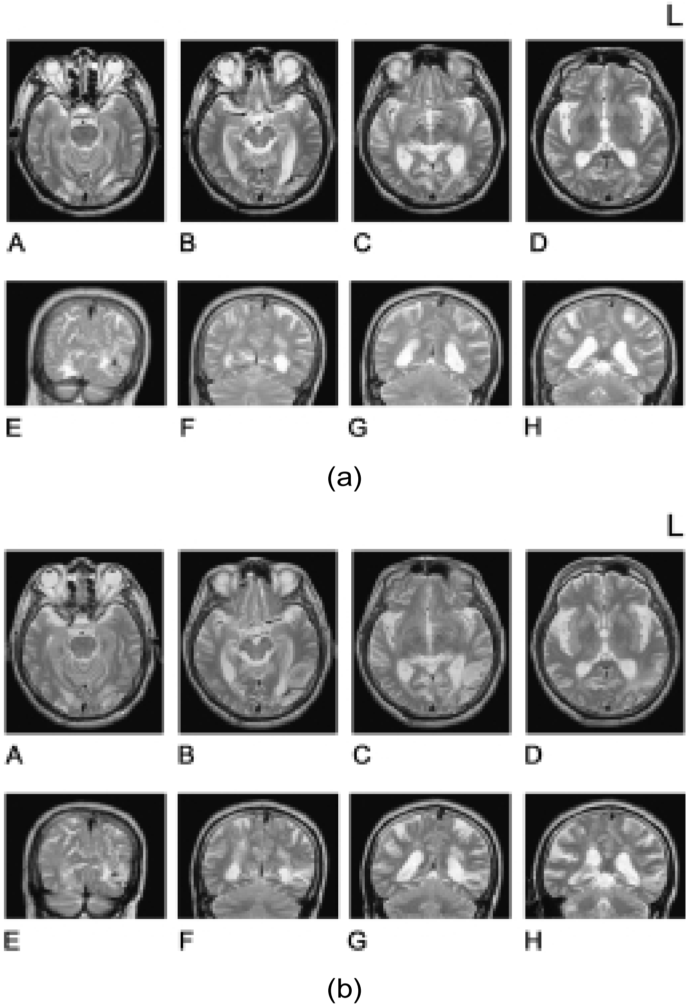

Fig. 2. a. MRI images at five months after the first infarction in patient 1. A high intensity area with marginal low intensity on T2-weighted axial (A-D) and coronal (E-H) images (1.5 Tesla, time of repetition [TR]/time of echo [TE] = 4000/81), suggesting a hemorrhagic infarction, was noted in the left middle and inferior occipital gyri. Another high intensity area was observed in the right basal occipital cortex (A, E). b. MRI images at eight days after the second infarction in patient 1. A high intensity area on T2-weighted axial (A-D) and coronal (E-H) images (1.5T, TR/TE = 4000/82 in axial images, 3500/81 in coronal images) was evident in the left posterior middle temporal gyrus that was located just anterior to the old lesion.

with paralexia of both kanji and kana. Recognition of orally spelled kanji characters and oral spelling of kanji characters were very poor, indicating character imagery deficits (impaired character recall). In spontaneous writing, literal paragraphia of kanji, a kind of homophone error, and substitution of kanji for the kana transcription were observed (Fig. 3). In all, his language profile was rated as alexia with agraphia for kanji (orthographic alexia with agraphia [26]) and minimal anomia mixed with the preceding pure alexia for kana. MRI administered eight days after onset showed a high intensity area on T2-weighted images in the left posterior middle temporal gyrus that extended deep to the posterior horn of the lateral ventricle (Fig. 2b). Adjacent to this lesion, a high intensity area with marginal low intensity was located posteriorly in the middle and inferior occipital gyri, suggesting the first stroke of a hemorrhagic infarction (Fig. 2b–A, B).

### *2.1.2. Patient 2*

A 60-year-old right-handed man, senior high school graduate and public official, noticed that he had difficulty reading kanji characters in a newspaper and recalling kanji characters when writing in January, 2007. Two weeks later, he consulted the department of neurosurgery at our hospital and was diagnosed as having a cerebral hemorrhage on MRI. He could read newspapers without difficulty by six weeks after onset. However, he noticed that he sometimes first read the second character in a kana character sequence. Because of persistent hypertension, he was introduced to our department. Neurological and neuropsychological examinations six weeks after onset showed agraphia for kanji due to impaired character recall. WAB two months post-onset revealed a slight writing impairment (Table 1). In spontaneous writing, he could not recall some kanji characters and omitted or added a kana character in some words. MRI administered 23 days after onset revealed a high intensity area on both T1- and T2-weighted images, suggesting a hemorrhage and surrounding edema, in the left posterior middle and inferior temporal gyri that extended subcortically to the posterior horn of the lateral ventricle (Fig. 4).

### 2.2. *Special neuropsychological tests*

Patient 1 underwent the following special neuropsychological tests (1), (2) and (3) [33] 23 days to four months after the first stroke and 10 to 11 days after the second stroke. Follow-up studies were conducted at six to 10 months and one year after the second stroke. He was also given Test (4) 22 days after the second stroke. Patient 2 was given these tests two to three months after onset. Reexamination was performed five to six months post-onset.

#### 2.2.1. *Test 1* (*reading and writing test*)

Reading of 100 single-character kanji and the kana transcription of kanji characters, and writing of the same 100 dictated kanji and kana [31]. Most of the kanji characters had two or more readings, i.e. *on*-readings and *kun*-readings. We read aloud the *kun*-reading of a kanji character, and the patient wrote it down in kanji or kana. All of the kanji characters are taught in the first three years of primary school in Japan.

#### 2.2.2. *Test 2* (*100 word reading test*)

Reading of 100 two-character kanji words, the corresponding 100 three-character hiragana words (high familiarity, described below) that were transcribed from the above kanji words, another set of 100 three-character hiragana words (low familiarity), and two sets of 100 three-character hiragana nonwords. The kana words were chosen from those with higher (hf) or lower (lf) familiarity based on how often the subject had seen, heard or used the word [17]. Mean familiarity values in auditory presentation assessed in a more recent study [2] were 5.98 for high familiarity words and 4.20 for low familiarity words on a seven-point rating scale. Two sets of kana nonwords were provided as follows: (a) (low association character) those combining kana symbols that had no association with each other [1] and (b) (changed character order) those changing the sequential order of characters in the above higher familiarity kana words.

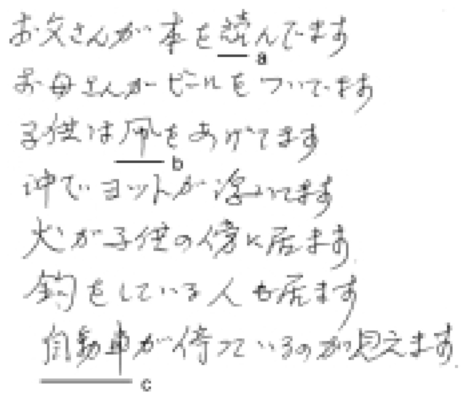

(A)

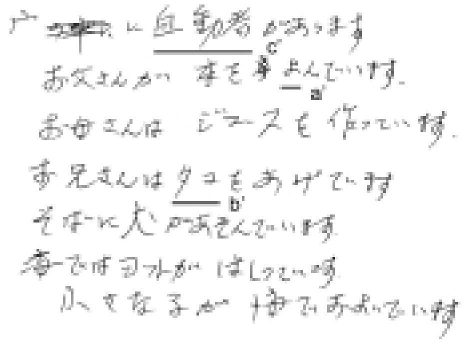

(B)

Fig. 3. Written output in the WAB test in patient 1. A. Five months after the first stroke. B. Eight days after the second troke. The patient was presented with a picture card and asked to write a story about what was going on in the picture within three minutes. Writing was normal in the first trial (A). In the second trial (B) after the left middle temporal gyrus infarction, there were fewer kanji characters and some kanji words were substituted for kana characters. a, a’: A kanji word was substituted for the hiragana (a cursive form of kana that is used for grammatical formatives) transcription after the second stroke. b, b’: A kanji word was substituted for the katakana (a square form of kana that is used primarily for representing loan words) transcription. c, c’: A kanji character in a three-character kanji word was substituted for another character that was phonologically the same, but orthographically different (homophone error), i.e. 自動車 ([jidousha], car) → 自動者 ([jidousha], nonword).

#### 2.2.3. *Test 3* (*multiple character reading test*)

Reading of single-character kana symbols, five-character kana words and nonwords, and the total or

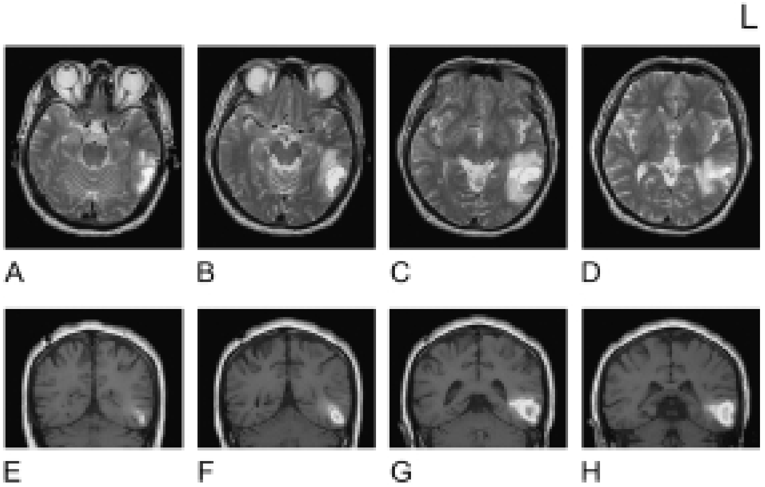

Fig. 4. MRI images at 23 days after onset in patient 2. A high intensity area on both T2-weighted axial (A-D) and T1-weighted coronal (E-H) images (1.5 T, TR/TE = 4000/81 in axial images, 600/14 in coronal images), suggesting a hemorrhage and surrounding edema, was localized in the left posterior middle and inferior temporal gyri.

partial kanji transcription of the five-character kana words (mixture of one to three kanji words and one to three kana characters untranscribed, e.g. お父さん [otou-san], father). The five-character kana words were selected from those with higher familiarity based on how often the subject had seen, heard or used the word [9]. Five-character kana nonwords were made by changing the sequential order of characters in the above five-character kana words.

*2.2.4. Test 4* (*visual discrimination test*) *[27]*

The materials were two sets of line drawings of slightly different lengths, sizes, shapes, spatial locations, and angles. The patient was asked whether the two stimuli drawn on a card were the same or different and, if different, to explain the difference.

## 3. Results

Figure 5 summarizes the % correct responses for each item in Tests 1 (reading and writing test), 2 (100 word reading test) and 3 (multiple character reading test).

### *3.1. Patient 1*

At one to four months after the first infarction in the occipital lobe, the patient showed deficits in reading single-character kana, five-character kana words and nonwords (Table 2). Letter-by-letter reading was noted in the five-character kana word and nonword reading tests. No word length effect was clearly observed (single-character, 1.56 sec/character; three-character word (hf), 4.30 sec/word; five-character word, 4.04 sec/word), probably because the reading test of five-character words was performed a week after the single-character kana and three-character kana word (hf) reading, so alexia had recovered to some extent. Kanji reading was also slightly impaired in the kanji-transcription of five-character kana words (III in Table 2). However, it took patient 1 more than twice as long to read kana words as to read kanji words in all the test items, which is not the case with normal controls [26]. This fact implies that alexia was more profound for kana.

In the second examination conducted 10 to 11 days after the second infarction in the posterior middle temporal gyrus (July 2005 in Table 2), an impairment in kanji writing was evident. Most of the error types were non- and partial responses that were due to impaired character recall (Table 3-B). Noteworthy is that the patient did rehearsals, moving the pencil over the paper before writing down a character, subsequently succeeding in recalling in six of 26 trials. To determine the effects of visual complexity, concreteness, familiarity (how often a person has seen or used a word) and frequency of writing single-character kanji, we divided the test characters into two groups (above or under a median) nearly equal in number: a more complex (more writing stroke sequences), concrete, familiar or frequent group and a less complex, concrete, familiar or frequent group [33]. Correct scores for the two groups were significantly different in complexity ($p < 0.0001$ by Fisher's exact method), frequency ($p = 0.0048$ by

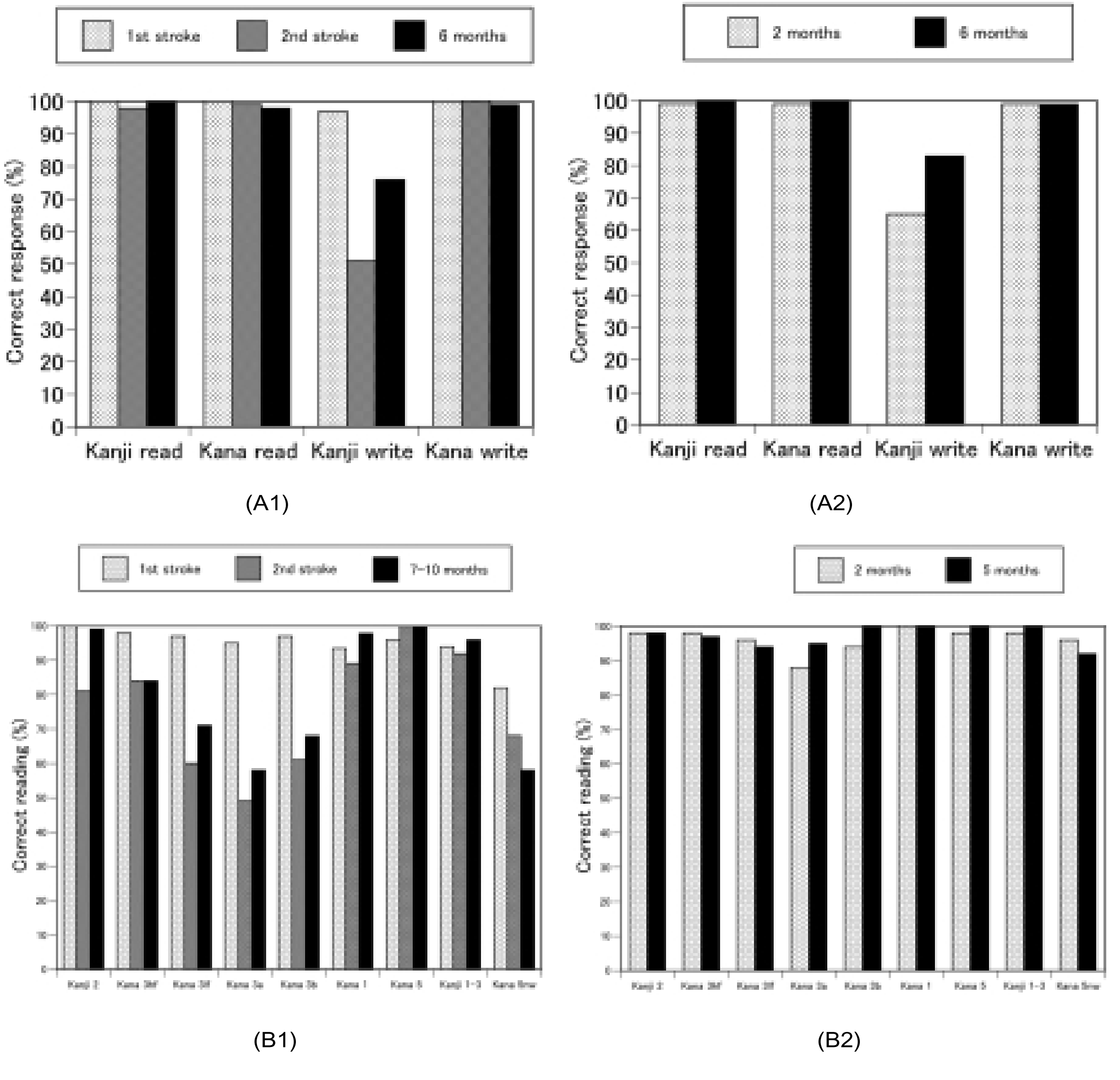

Fig. 5. Percent correct responses in Test 1 (reading and writing test) (A1, Patient 1; A2, Patient 2) and in Tests 2 (100 word reading test) and 3 (multiple character reading test) (B1, Patient 1; B2, Patient 2). Abbreviations. Kanji 2, 2-character kanji words; Kana 3hf, 3-character kana words (high familiarity); Kana 3lf, 3-character kana words (low familiarity); Kana 3a, 3-character kana nonwords (a) (low character association); Kana 3b, 3-character kana nonwords (b) (changed character order); Kana 1, single-character kana; Kana 5, 5-character kana words; Kanji 1–3, kanji transcription of 5-character kana words; Kana 5nw, 5-character kana nonwords.

Fisher's exact method) and familiarity ($p < 0.0001$ by Fisher's exact method), i.e. less complex, more frequent and more familiar characters were written more easily. Two-character kanji reading and three-character kana word and nonword reading were also disturbed. Kana reading errors consisted of phonological and phonological/visual (one or more characters in a word were substituted for another character that was visually similar to the correct answer) responses (Table 3-A). A word length effect became evident (single-character, 1.63 sec/character; three-character word (hf), 5.11 sec/word; five-character word, 5.74 sec/word). High-familiarity kana words were read better than low-familiarity kana words ($p = 0.0006$ by Fisher's exact method; familiarity effect), and kana words (hf) were read better than kana nonwords (a) (low association character) and (b) (changed character order) ($p < 0.0005$ by Fisher's exact method; lexicality effect). In Test 4 (visual discrim-

Table 2
Reading and writing test results

| Patient | Patient 1 | | | Patient 2 | |
|---|---|---|---|---|---|
| Time | Jan-May 2005 | July 2005 | Jan-May 2006 | Mar-Apr 2007 | June–July 2007 |
| (after onset, mo.) | 1–4[a] | 1[b] | 6–10[c] | 2–3 | 5–6 |
| I. Reading and writing (100 single-character kanji and kana transcription), % correct (time) | | | | | |
| Kanji reading | 100 (1 min) | 98 (2 min)[d] | 100 (2 min) | 99 (1 min) | 100 (1 min) |
| Kana reading | 100 (1 min) | 99 (5* min)[e] | 98 (5* min) | 99 (1 min) | 100 (1 min) |
| Kanji writing | 97 (8 min) | 51* (30* min) | 76* (13 min) | 65* (7 min) | 83* (7 min) |
| Kana writing | 100 (6 min) | 100 (7 min) | 99 (7 min) | 99 (5 min) | 99 (5 min) |
| II. Reading (100 two-character kanji words, three-character kana words and three-character kana nonwords), % correct (time) | | | | | |
| Kanji words | 100 (3* min) | 81* (5* min) | 99 (4* min) | 98 (1 min) | 98 (1 min) |
| Kana words (hf)[f] | 98 (7* min) | 84* (8* min) | 84* (7* min) | 98 (1 min) | 97 (1 min) |
| Kana words (lf)[f] | 97 (4 min) | 60* (5* min) | 71* (7* min) | 96 (2 min) | 94 (2 min) |
| Kana nonwords (a)[f] | 95 (4 min) | 49* (7* min) | 58* (8* min) | 88 (2 min) | 95 (2 min) |
| Kana nonwords (b)[f] | 97 (4* min) | 61* (6* min) | 68* (7* min) | 94* (2 min) | 100 (2 min) |
| III. Reading (46 single-character kana, 50 five-character kana words and five-character kana nonwords), % correct (time) | | | | | |
| Single-character (/46) | 93* (1* min) | 89* (1* min) | 98 (1* min) | 100 (1 min) | 100 (1 min) |
| Five-character words (/50) | 96* (3* min) | 100 (4* min) | 100 (5* min) | 98 (1 min) | 100 (1 min) |
| Kanji transcription (/50) | 94* (1* min) | 92* (2* min) | 96* (2* min) | 98 (1 min) | 100 (1 min) |
| Five-character nonwords (/50) | 82* (3 min) | 68* (5* min) | 58* (7* min) | 86 (1 min) | 92 (1 min) |
| IV. Visual discrimination test, % correct | | | | | |
| Line length (/12) | | 92 | | 100 | |
| Circle size (/12) | | 92 | | 100 | |
| Parallel line or no (/12) | | 92* | | 100 | |
| Circle-oval (/12) | | 67* | | 83* | |
| Square-rectangle (/12) | | 92 | | 92 | |
| Point location (/12) | | 100 | | 100 | |
| Distortion of a square (/12) | | 58* | | 100 | |
| Angle (/12) | | 83* | | 100 | |

[a]Test I was performed in May, 2005 (four months post-onset) and Tests II and III were performed from January to February, 2005 (one to two months post-onset). In Tests II and III, reading of kana words (lf), kana nonwords (a) (low association character) and (b) (changed character order), five-character kana words and nonwords, and kanji transcription of five-character kana words were conducted a week after single-character kana, kanji word and kana word (hf) reading.
[b]Ten to 11 days after the second infarction in the posterior middle temporal gyrus.
[c]Test I was performed in January, 2006 (six months after the second infarction) and Tests II and III were performed in March through May, 2006 (eight to ten months after the second infarction).
[d]Errors consisted of one visual (changing the kanji reading to the reading of another kanji with a visual resemblance to the correct answer) and one unrelated (changing a kanji to another kanji that has no visual or semantic similarity to the correct answer) response.
[e]The error was a phonological response (one or more characters in a kana word were substituted for other kana, i.e. phonemic paralexia).
[f]Kana words consisted of higher (hf) and lower (lf) familiarity words. Kana nonwords were made by combining kana symbols that had no association with each other (a) (low association character) and by changing the sequential order of characters in the above higher familiarity kana words (b) (changed character order).
*More than 2SD above (for time) or below (for score) the normal mean [27,32,33].

ination test), he achieved low scores when discriminating the following visual stimuli (more than 2SD below the normal mean [27]): parallel line or not, circle or oval, distorted square or not, and angle difference (IV in Table 2).

In a follow-up study conducted six to ten months after the second infarction (Jan-May 2006 in Table 2), kanji reading recovered to within the normal range and kanji writing was considerably improved, although kanji character recall remained impaired. Reading of single-character kana, three-character kana words (lf), and three-character kana nonwords (a) (low association character) and (b) (changed character order) were generally improved, but reading of five-character nonwords was somewhat worse. Also, a word length effect was still observed (single-character, 1.30 sec/character; three-character word (hf), 4.47 sec/word; five-character word, 6.24 sec/word).

A reexamination one year after the second stroke revealed an improvement in three-character kana reading (kana words (hf), from 84 to 96; kana nonwords (a) (low association character), from 58 to 71) but no change in five-character kana (kana words, from 50 to 46; kana nonwords, from 29 to 31). Conversely, kanji

Table 3A
Types of reading error in two-character kanji word, three-character kana word (hf) and three-character kana nonword (a) (low association character) test (values are the number of errors)

| Patient | Patient 1 | | Patient 2 |
|---|---|---|---|
| Time | Jan-May 2005 | July 2005 | Mar 2007 |
| (after onset, mo.) | 1–4 | 1 | 2 |
| Two-character kanji word ($n = 100$) | | | |
| Non-response | 0 | 6 | 0 |
| Partial[a] | 0 | 8 | 2 |
| Unrelated[a] | 0 | 5 | 0 |
| Total errors | 0 | 19 | 2 |
| Three-character kana word (high familiarity) ($n = 100$) | | | |
| Phonological[b] | 2 | 13 | 1 |
| Phonological/visual[b] | 0 | 3 | 1 |
| Total errors | 2 | 16 | 2 |
| Three-character kana nonword (a) (low association character) ($n = 100$) | | | |
| Phonological[b] | 3 | 35[c] | 9[c] |
| Phonological/visual[b] | 2 | 16 | 3 |
| Total errors | 5 | 51 | 12 |

[a]Partial response: one symbol of a two-character kanji word could not be read. Unrelated response: changing the reading of a kanji word to the reading of another kanji word that has no visual or semantic similarity to the correct answer, e.g. 支配 ([shihai], control) → 反則 ([hansoku], foul). Two of five errors in patient 1 (July 2005, 11 days after the second stroke) were perseveration responses.
[b]Phonological response: one or more characters in a kana word or nonword were substituted for other kana (phonemic paralexia), e.g. とかい ([tokai], city) → とおい ([tooi], far). Phonological/visual response: changing one character in a word to another visually similar to the target character, e.g. もはん ([mohan], example) → もほん ([mohon], nonword).
[c]Five of 35 errors in patient 1 and Five of nine errors in patient 2 occurred at the level of vowel conversion in a consonant-vowel mora, e.g. つろも [tsu-ro-mo] → つろむ [tsu-ro-mu].

Table 3B
Types of writing error in the 100 single-character kanji test (values are the number of errors)

| Patient | Patient 1 | | Patient 2 |
|---|---|---|---|
| Time | Jan-May 2005 | July 2005 | Mar 2007 |
| (after onset, mo.) | 1–4 | 1 | 2 |
| Single-character kanji ($n = 100$) | | | |
| Non-response | 1 | 42 | 34 |
| Partial* | 0 | 4 | 1 |
| Constructional* | 1 | 0 | 0 |
| Visual* | 1 | 0 | 0 |
| Unrelated* | 0 | 3 | 0 |
| Total errors | 3 | 49 | 35 |

*Partial response: the patient wrote a component of a character correctly, but did not write the other components. Constructional response: the omission or addition of a component of a kanji, e.g. 牛 ([ushi], cow) → 午 ([go], noon). Visual response: substitution of another kanji visually similar to the target character, e.g. 軽 ([karui], light) → 転 ([korogaru], roll). Unrelated response: substitution of another kanji that has no visual or phonological similarity to the correct answer, e.g. 来 ([kuru], come) → 今 ([ima], now).

writing was somewhat worse (correct response, from 76 to 69). At this time, naming was normal in the WAB test (object naming, 60/60).

### 3.2. Patient 2

In Test 1 (reading and writing test), agraphia of kanji was prominent (Mar-Apr 2007 in Table 2). Nearly all the writing errors were non-responses due to impaired character recall (Table 3-B). The writing impairment was influenced by complexity ($p = 0.0001$ by Fisher's exact method), frequency ($p = 0.035$ by Fisher's exact method) and familiarity ($p = 0.037$ by Fisher's exact method). Patient 2 read kanji and kana in Tests 2 (100 word reading test) and 3 (multiple character reading test) as fast as normal controls, but misread kana nonwords (phonemic paralexia). Kana reading errors consisted of phonological and phonological/visual responses (Table 3-A). Five of nine phonological responses in kana nonword errors occurred at the level of vowel conversion in a consonant-vowel mora (see the footnote to Table 3-A). Kana words (hf) were read better than kana nonwords (a) (low association character) ($p = 0.0055$ by Fisher's exact method). He did not exhibit letter-by-letter reading or a word-length effect (singlecharacter, 0.47 sec/character; three-character word (hf), 1.16 sec/word; five-character word, 1.54 sec/word). In Test 4 (visual discrimination test), he achieved a lower score in circle-oval discrimination.

In a reexamination five to six months after onset, kanji writing improved but was still slightly impaired, whereas kana nonword reading recovered to within the normal range (June–July 2007 in Table 2).

### 3.3. *Neuroimaging study*

Patient 1 underwent single photon emission computed tomography with a $^{99m}$Tc-ethylcysteinate dimer (ECD-SPECT) five months after the first infarction and 24 days after the second infarction, and patient 2 was given the scans 10 weeks after onset. SPECT data were transformed into the Analyze format and were normalized, smoothed and corrected for inter-laboratory differences with a three-dimensional conversion map with the easy Z-score Imaging System (eZIS) version 2 [19]. In this system, realignment, spatial normalization and smoothing were essentially the same as those of Statistical Parametric Mapping (SPM [8]) Version 1999, and the statistical significance was determined with a two-sample $t$-test after adjusting the global blood flow to 50 ml/min/dl for one patient vs. group (normal control) analysis. The data were compared with those of a normal subject database of the same generation and gender at the National Center of Neurology and Psychiatry, Tokyo ($n = 20$ for over 70 year-old men and $n = 18$ for 60–69 year-old men). Areas showing a significant decrease in cerebral blood flow (uncorrected $p < 0.001$) were rendered on standard brain surface images (Fig. 6). In patient 1, the first scan revealed significantly reduced blood flow in the left middle and inferior occipital gyri and the right posterior fusiform gyrus (Area 18/19). In the second scan, hypoperfusion of the left middle and inferior occipital gyri extended to the posterior middle temporal gyrus. The angular gyrus (Area 39) and the mid fusiform/inferior temporal gyri (Area 37) were spared. In patient 2, blood flow reduction was restricted to the left posterior middle temporal gyrus and the cerebellar vermis.

## 4. Discussion

Patient 1 first presented with alexia more impaired for kana and right hemianopia after an infarction in the left middle and inferior occipital gyri and the right basal occipital cortex. Six months later when alexia recovered to within the normal range, he suffered a second infarction in the left posterior middle temporal gyrus that was continuous to the first lesion. This time alexia with agraphia for kanji occurred, mixed with worsened alexia for kana. Over the following six months, kanji reading recovered while agraphia for kanji remained, together with alexia for kana. On the other hand, patient 2 showed alexia with agraphia for kanji in the acute phase and agraphia for kanji remained, together with minor alexia, particularly for kana nonwords. At six months after onset kana reading became normal, whereas kanji agraphia persisted. Therefore, we can designate the condition at this stage as pure agraphia for kanji.

The clinical profiles of these patients raise two important points. First, alexia with agraphia for kanji that resolved to agraphia for kanji occurred in a focal lesion in the left posterior middle and inferior temporal gyri. Patient 1 had damage to the right basal occipital cortex from the first stroke and it is conceivable that this lesion affected his reading performance. However, a left occipital gyri lesion is enough to give rise to pure alexia for kana [27].

Second, alexia for kana became evident or worse in a lesion of the posterior middle and inferior temporal gyri. In patient 1 both kana words and kana nonwords were affected, whereas in patient 2 alexia was limited to kana nonwords.

### 4.1. *Agraphia for kanji*

Concerning the first point, the common lesion of the posterior middle temporal gyrus was close to the posterior inferior temporal cortex (mid fusiform/inferior temporal gyri, Area 37) or VWFA [5], damage to which results in alexia with agraphia for kanji or orthographic alexia with agraphia [26]. Probably, because the mid fusiform/inferior temporal gyri were functionally affected by the middle temporal gyrus lesion, the patient showed "alexia" in addition to agraphia for kanji in the acute phase. The characteristics of agraphia were impaired recall of kanji orthography, which was influenced by character complexity, frequency and familiarity. These features are similar to those of the writing impairment in alexia with agraphia for kanji from a posterior inferior temporal cortex lesion: a patient with alexia with agraphia for kanji we previously reported [26,31,32] showed severe impairment of kanji writing (correct response, kanji 12/100; kana 77/100), characterized by a familiarity effect ($p = 0.0139$ by Fisher's exact method) and a trend towards a complexity effect

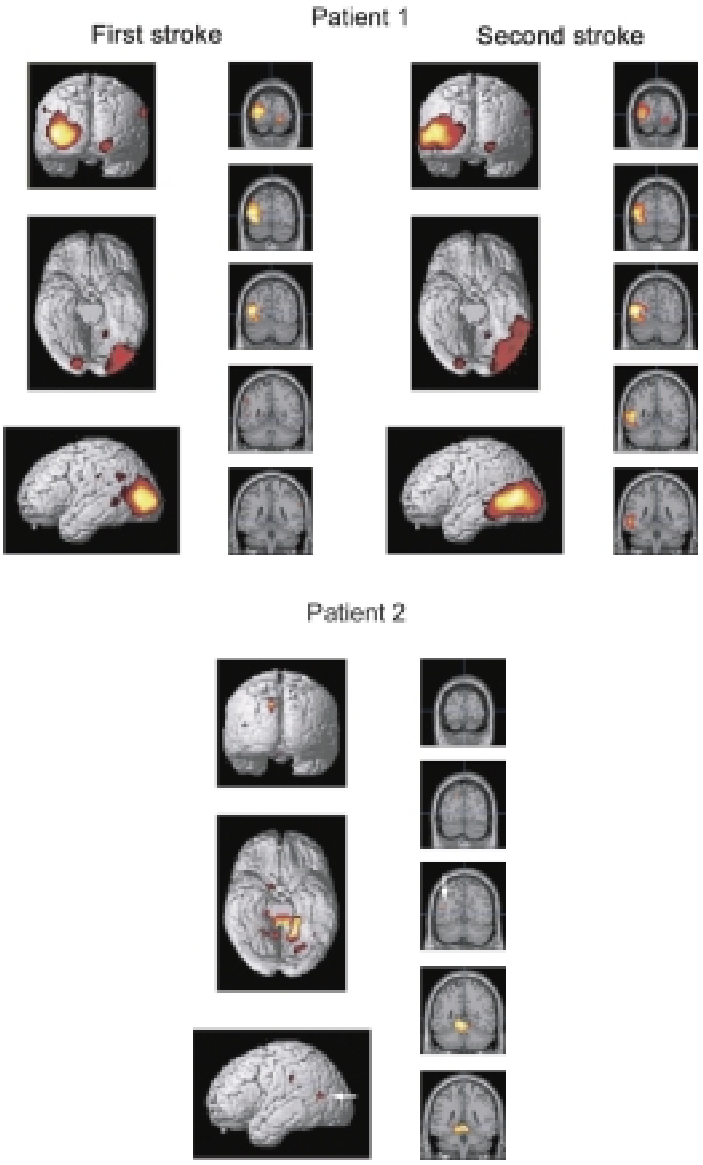

Fig. 6. $^{99m}$Tc-ECD-SPECT images in patient 1 at five months after the first infarction in the occipital lobe and 24 days after the second infarction in the posterior middle temporal gyrus, and in patient 2 at two months after onset. Patient data were compared with those of normal volunteers of the same generation and gender with a two-sample $t$-test of SPM 99. Coronal planes are at the level of the calcarine sulcus and lateral occipital gyri (upper two, $y = -90$, $-80$ mm), the angular gyrus (middle, $y = -70$ mm in patient 1 and $-64$ mm in patient 2), and the mid fusiform and temporal gyri (lower two, $y = -50$, $-40$ mm). A vertical line denotes the z-axis ($x = 0$) and the horizontal line denotes the x-axis ($z = 0$) in the Montreal Neurological Institute (MNI) coordinate space. In patient 1, reduced blood flow was noted in the left middle and inferior occipital gyri and the right posterior fusiform gyrus (Area 18/19) after the first stroke. After the second stroke, the area of hypoperfusion extended from the lateral occipital gyri to the posterior middle temporal gyrus. In patient 2, a restricted area of hypoperfusion was observed in the left posterior middle temporal gyrus (arrow).

($p = 0.0689$ by Fisher's exact method) at 15 months after onset of the disease (unpublished data). One of the main differences is that alexia of kanji in a posterior middle temporal gyrus lesion is transient, whereas that in a mid fusiform/inferior temporal gyri lesion is persistent [32]. This fact implies that in agraphia for kanji in a posterior middle temporal gyrus lesion, visual images of words are intact, but access to the images or output from the image storage is interrupted.

Previous reports on lexical agraphia or its Japanese equivalent, pure agraphia for kanji, also involved the left middle temporal gyrus [7,34], but there have been few reported patients with an isolated lesion in the middle temporal gyrus. Only one patient reported by Yokota et al. [39] had a relatively limited area of hemorrhage in the posterior middle temporal gyrus that extended deep to the lateral ventricle, but this patient had angular gyrus involvement in the acute phase. Our patients reveal that a lesion of the posterior middle temporal gyrus alone can cause agraphia for kanji.

Functional imaging studies on writing revealed activation of the left posterior inferior temporal cortex (Area 37) in writing English words ($x = -42$, $y = -54$, $z = -12$ in the Montreal Neurological Institute [MNI] coordinate space [4]) and kanji words ($x = -50$, $y = -56$, $z = -12$ [38]; $x = -43$, $y = -58$, $z = -9$ [21]). It should be noted, however, that the peak activation locus was a little higher than when reading European words ($x = -42$, $y = -63$, $z = -15$ [6]; $x = -46$, $y = -56$, $z = -22$ [36]) and kanji words ($x = -44$, $y = -54$, $z = -22$ [29]), and was identified more in the inferior temporal gyrus than in the mid fusiform/inferior temporal gyri or VWFA. This difference in peak activation may reflect the fact that agraphia (without alexia) for kanji results from damage to the posterior middle/inferior temporal gyri, whereas alexia with agraphia for kanji results from damage to the mid fusiform/posterior inferior temporal gyri.

We previously hypothesized that visual images of words and letters (orthographic lexicon) are stored in the posterior inferior temporal cortex and go upward via the subcortical area in the angular gyrus to the superior parietal lobule, and then travel further to the frontal motor and premotor area (orthographic route in writing, described in the Introduction; Fig. 1) [28,30]. According to this hypothesis, damage to the posterior middle/inferior temporal gyri interferes with the phonological access from the phonological lexicon (posterior superior temporal gyrus) to the orthographic lexicon (illustrated with “x” on “c; P→ O” route in Fig. 1), or interrupts visual image transfer along the orthographic route from the posterior inferior temporal cortex to the parietal lobe (illustrated with “x” on “b; O → AG” route in Fig. 1), which may result in agraphia for kanji or lexical agraphia.

### *4.2. Alexia for kana*

In patient 1, kanji reading was also slightly impaired after the first infarction. Thus, we can safely state that the patient had pure alexia that affected kana more than kanji (pure alexia more impaired for kana). We assumed that pure alexia for kana became more profound as the lesion involved extensive areas in the ventral and dorsal side of the occipital lobe [27,33]. Since the lesion extended continuously from the middle and inferior occipital gyri to the middle temporal gyrus, it is conceivable that kana alexia worsened. On the other hand, patient 2 showed that kana nonword reading was minimally disturbed in an isolated lesion in the left posterior middle and inferior temporal gyri, although single-character kana reading was preserved and letter-by-letter reading was not observed. In addition, more than half of the reading errors were due to inappropriate vowel conversion in a consonant-vowel mora. These findings suggest that the deficit lay in the stage of grapheme-to-phoneme conversion, rather than in the stage of letter identification.

The combination of agraphia for kanji and alexia for kana was reported in a temporo-parietal area lesion [20] and inferior occipital cortex lesions [27,33], as in our patient 1. In the case of the temporo-parietal area lesion, unlike our patient 2, kana alexia was evident in kana “real words.” Damage to the inferior occipital cortex causes pure alexia for kana sometimes accompanied by minimal kanji agraphia, although patient 1 with the inferior occipital cortex lesion did not develop kanji agraphia after the first stroke. Since patient 2 did not show letter-by-letter reading or a word-length effect, he did not sustain pure alexia. Patient 2 is more similar to the original case of lexical agraphia in European counties that was accompanied by phonological alexia (selective impairment of nonword reading) in a parieto-occipital region injury [3], although the kana nonword reading impairment in patient 2 was too slight to call this symptom phonological alexia.

It is yet to be determined whether kana nonword alexia results from damage to the posterior middle temporal gyrus or reflects the involvement of the surrounding angular and lateral occipital gyri. According to our hypothesis [26,29], sequential processing from letter identification to grapheme-phoneme conversion is performed in a putative route from the medial to lateral occipital gyri to the posterior superior temporal gyrus via the temporo-parietal junction (deep perisylvian cortex, Area 40/22). This phonological processing route in reading (see Fig. 3 in Sakurai [26]) may be influenced by a posterior middle temporal gyrus lesion.

## 5. Conclusion

Damage to the posterior inferior temporal cortex or VWFA does not yield pure alexia or pure agraphia, but rather alexia with agraphia for kanji or orthographic alexia with agraphia [26]. If afferent access via visual input to this area is interrupted more medially in the mid fusiform gyrus, pure alexia for words [33] may occur. If afferent access via auditory input to this area or efferent output from the area is interrupted in the posterior middle/inferior temporal gyri, agraphia for kanji or lexical agraphia may occur.

The present study demonstrates that agraphia for kanji arises from an isolated lesion in the left posterior middle temporal gyrus. If the adjacent mid fusiform/inferior temporal gyri (Area 37) are spared, alexia for kanji is transient. A remaining problem is whether damage to the posterior middle temporal gyrus causes pure agraphia for kanji or agraphia for kanji with alexia for kana. Although patient 2 had pure agraphia for kanji six months after onset, it is unknown whether accompanying kana paralexia was caused by the posterior middle temporal gyrus lesion or the surrounding area. Further study is required to solve this problem.
